# Inhibitory Effects of Cucurbitane-Type Triterpenoids from *Momordica charantia* Fruit on Lipopolysaccharide-Stimulated Pro-Inflammatory Cytokine Production in Bone Marrow-Derived Dendritic Cells

**DOI:** 10.3390/molecules26154444

**Published:** 2021-07-23

**Authors:** Thao Quyen Cao, Nguyen Viet Phong, Jang Hoon Kim, Dan Gao, Hoang Le Tuan Anh, Viet-Duc Ngo, Le Ba Vinh, Young Sang Koh, Seo Young Yang

**Affiliations:** 1 Drug Research and Development Center, College of Pharmacy, Daegu Catholic University, Gyeongsan-si 38430, Korea; caothaoquyen@tdtu.edu.vn; 2 Institute of Pharmaceutical Research and Development, College of Pharmacy, Wonkwang University, Iksan 54538, Korea; 3 Institute of Marine Biochemistry (IMBC), Vietnam Academy of Science and Technology (VAST), Hanoi 100000, Vietnam; ngvietphong@gmail.com (N.V.P.); vinhrooney@gmail.com (L.B.V.); 4 Department of Herbal Crop Research, National Institute of Horticultural and Herbal Science, RDA, Eumseon 27709, Korea; oasis5325@gmail.com; 5 College of Pharmacy, Chungnam National University, Daejeon 34134, Korea; dangao@gmail.com; 6 Center for Research and Technology Transfer, VAST, Hanoi 100000, Vietnam; hoangletuananh@hotmail.com (H.L.T.A.); ngovietduc@tdt.edu.vn (V.-D.N.); 7 Department of Medicine, School of Medicine, Jeju National University, 102 Jejudaehakno, Jeju 63243, Korea; yskoh7@jejunu.ac.kr; 8 Department of Pharmaceutical Engineering, Sangji University, Wonju 26339, Korea

**Keywords:** *Momordica charantia*, bioactive compound, anti-inflammatory effect, triterpenoid

## Abstract

The bitter melon, *Momordica charantia* L., was once an important food and medicinal herb. Various studies have focused on the potential treatment of stomach disease with *M*. *charantia* and on its anti-diabetic properties. However, very little is known about the specific compounds responsible for its anti-inflammatory activities. In addition, the in vitro inhibitory effect of *M*. *charantia* on pro-inflammatory cytokine production by lipopolysaccharide (LPS)-stimulated bone marrow-derived dendritic cells (BMDCs) has not been reported. Phytochemical investigation of *M*. *charantia* fruit led to the isolation of 15 compounds (**1**−**15**). Their chemical structures were elucidated spectroscopically (one- and two-dimensional nuclear magnetic resonance) and with electrospray ionization mass spectrometry. The anti-inflammatory effects of the isolated compounds were evaluated by measuring the production of the pro-inflammatory cytokines interleukin IL-6, IL-12 p40, and tumor necrosis factor α (TNF-α) in LPS-stimulated BMDCs. The cucurbitanes were potent inhibitors of the cytokines TNF-α, IL-6, and IL-12 p40, indicating promising anti-inflammatory effects. Based on these studies and in silico simulations, we determined that the ligand likely docked in the receptors. These results suggest that cucurbitanes from *M*. *charantia* are potential candidates for treating inflammatory diseases.

## 1. Introduction

Inflammation is a beneficial host response to an external challenge or cellular injury that leads to the release of inflammatory mediators, finalizing the restoration of tissue function and structure [1]. Once activated, inflammatory cells secrete increased amounts of nitric oxide (NO), prostaglandin E_2_, and cytokines (e.g., interleukin (IL)-1β, IL-6, IL-12, and tumor necrosis factor-α (TNF-α)) to initiate the inflammatory process. These pro-inflammatory mediators can also serve as targets to disrupt this mechanism therapeutically [2]. Inflammation is subclassified into acute and chronic inflammation. Acute inflammation is a rapid response in the early stage of inflammation that typically lasts from minutes to a few days. It is usually beneficial for the host to initiate healing and promote tissue recovery. Once acute inflammation fails to resolve the damage, inflammation will progress to the chronic stage—a low-grade inflammation that persists for weeks, months, or even years [3]. The World Health Organization (WHO) ranks chronic diseases as the greatest threat to human health. Chronic inflammation is related to many human diseases, including diabetes, rheumatoid arthritis, atherosclerosis, asthma, neurodegenerative disorders, and cancer [3,4].

Natural products are rich sources for drug discovery, and the development of active components from medicinal herbs continues to be an important source of therapeutic agents for chronic inflammation [5]. The bitter gourd (*Momordica charantia* L.) belongs to the family Cucurbitaceae and has long been used in foods and medicines [6]. Bitter gourd possess anti-diabetic [6], anti-inflammation [7], anti-oxidant [8], anti-viral [8], anti-cancer [9], and anti-hyperlipidemic [10] effects. Phytochemical investigations revealed that cucurbitane-type triterpenes are the major subclass of compounds in *M*. *charantia* [7,9], and more than 270 cucurbitane-type triterpenoids have been isolated from plant organs with various pharmaceutical effects [11,12,13,14]. For instance, xuedanencins G and H isolated from the tubers of *Hemsleya penxianensis* were cytotoxic with IC_50_ values of 1.82 and 2.45 μM, respectively [14]. Cucurbitacin B isolated from *Hemsleya endecaphylla* had potent anti-HIV-1 effects in C8166 cells (EC = 0.09 μg/mL), with a selectivity index of 16.7 [15]. Cucurbitane-type triterpenoids from *M*. *charantia* reduced NO production with IC_50_ values of 11.3–29.1 μM [16].

Our continued efforts to study biologically active compounds isolated from medicinal herbs led to the isolation of 15 cucurbitane-type terpenoids (**1**–**15**) from *M*. *charantia* fruit. Here, we report the isolation, structure elucidation, and in vitro and in silico anti-inflammatory activities of the isolated metabolites in lipopolysaccharide (LPS)-stimulated bone marrow-derived dendritic cells (BMDCs).

## 2. Results and Discussion

**Figure 1:**
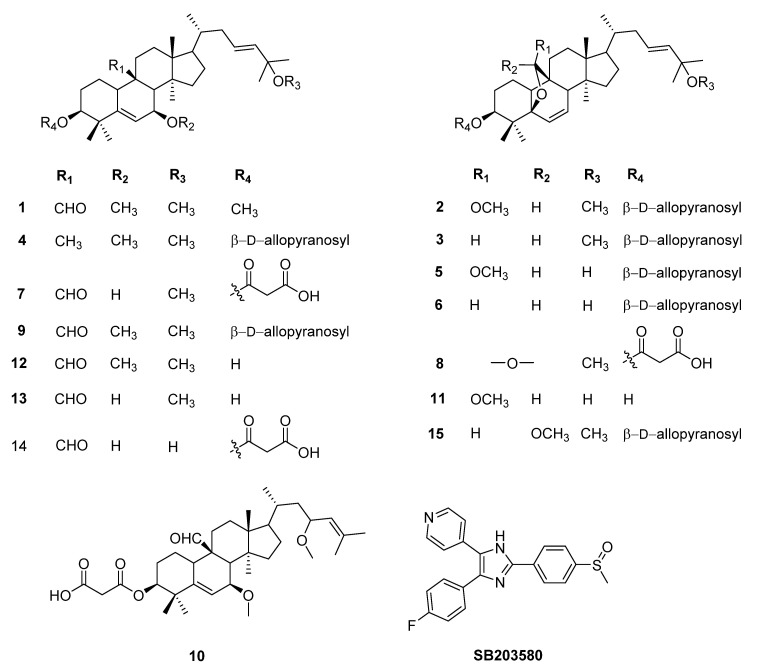
The isolated compounds from *M. charantia* (**1**–**15**) and **SB203580**.

To characterize the bioactive metabolites responsible for the anti-inflammatory effects, efficient chromatographic separation techniques enabled the isolation of 15 compounds (**1**–**15**) from *M*. *charantia*. (Figure 1), which were identified as 3,7,25-trimethoxycucurbita-5,23(*E*)-dien,19-al (**1**) [17], goyaglycoside-d (**2**) [18], momordicoside G (**3**) [19], karaviloside II (**4**) [20], goyaglycoside-b (**5**) [18], momordicoside F_2_ (**6**) [19], 3-[(5-formyl-7β-hydroxy-25-methoxycucurbita-5,23-dien-3-yl)oxy]-3-oxopropanoic acid (**7**) [21], 3-[(25-*O*-methylkaravilagenin d-3-yl)oxy]-2-oxoacetic acid (**8**) [21], 7β,25-dimethoxycucurbita-5(6),23(*E*)-dien-19-al 3-O-β-D-allopyranoside (**9**) [22], 3-[(5-formyl-7β-methoxy-7,23*S*-dimethoxycucurbita-5,23-dien-3-yl)oxy]-3-oxopropanoic acid (**10**) [21], (19*R*,23*E*)-5β,19-epoxy-19-methoxycucurbita-6,23-diene-3β,25-diol (**11**) [9], (23*E*)-3β-hydroxy-7β,25-dimethoxycucurbita-5,23-dien-19-al (**12**) [17], 3β,7β,25-trihydroxycucurbita-5,23(*E*)-dien-19-al (**13**) [17], 3-[(5-formyl-7β,25-dihydroxymethoxycucurbita-5,23-dien-3-yl)oxy]-3-oxopropanoic acid (**14**) [21], and 19*S*, 25-dimethoxy-5,23-dien-5β,19-epoxycucurbitane-3-*O*-β-allopyranoside (**15**) [18], by comparison of their spectral data with values reported in the literature.

Compound **1** was obtained as a white amorphous powder, with [α]_D_^20^ = −77.8 (*c* = 0.2, MeOH). The infrared (IR) spectrum of **1** showed strong absorption by hydroxyl (3398 cm^−1^) and ketone (1730 cm^−1^) groups, and a distinctive absorption band for an olefinic group (1066 cm^−1^). Using high-resolution electrospray ionization mass spectrometry (HR-ESI-MS), the molecular formula was determined to be C_33_H_54_O_4_ from the positive-ion [M + Na]^+^ at *m*/*z* 537.3918. To our knowledge, this is the first report of the nuclear magnetic resonance (NMR) assignment of this compound. The NMR spectra of **1** indicated a triterpenoid, one of the major components of *M*. *charantia*. The ^1^H-NMR spectrum of **1** showed seven tertiary methyl groups at *δ*_H_ 1.07, 1.15, 1.25, 1.43, and 1.50 (s, 3H) and 1.56 (s, 6H); three proton resonances at *δ*_H_ 5.71 (br m, H-23 and H-24) and 6.26 (d, *J* = 4.8 Hz, H-6); and three methoxy groups at *δ*_H_ 3.58 (s, 3H, H-3) and 3.45 (s, 6H, H-7 and H-25). The ^13^C-NMR spectrum together with the HMQC data showed 33 carbon signals, four olefinic carbons (*δ*_C_ 146.1, C-5; 124.5, C-6; 128.5 C-23; and 137.1, C-24), and one ketone (*δ*_C_ 208.0, C-19), including three methoxy at *δ*_C_ 56.1, 51.2, and 50.3. The NMR data of **1** were very similar to those of 3β,7β,25-trihydroxycucurbita-5,23(*E*)-dien-19-al, except for the presence of three methoxy groups at C-3, C-7, and C-25 [17]. Detailed analyses of the ^1^H-^1^H COSY, HMQC, and HMBC data confirmed the planar structure of **1**, which was identified as 3,7,25-trimethoxycucurbita-5,23(*E*)-dien,19-al. Previously, compound **1** has been produced by the methanolysis reaction [17].

### 2.1. Inhibition of IL-6 Expression

IL-6 is a pleiotropic, pro-inflammatory cytokine produced by various cell types, including lymphocytes, monocytes, and fibroblasts. In joints, the major source of IL-6 is from synovial fibroblasts, with some released by activated macrophages and lymphocytes [23]. Excess IL-6 production contributes to the anemia of chronic disease, which is common in active rheumatoid arthritis, by increasing hepcidin production and inducing thrombocytosis via increased megakaryocyte differentiation [24]. Furthermore, IL-6 may prompt synovial fibroblast differentiation and osteoclast activation, contributing to pannus formation and cartilage and bone destruction [25]. Dysregulation of IL-6 may explain some of the common clinical manifestations associated with active rheumatoid arthritis, including fever, weight loss, fatigue, and poor appetite [23].

To examine the inhibitory effects of *M*. *charantia* on IL-6 expression, LPS-stimulated BMDCs were treated with isolated metabolites **1**–**15**. As Table 1 shows, all of the isolated cucurbitane-type triterpenoids inhibited IL-6 production with IC_50_ values of 0.028–1.962 μM, while the positive control SB203580 had an IC_50_ of 5.000 μM. Compounds **3**, **4**, **6**, **11**, and **12** had dramatic effects, with IC_50_ values of 0.245, 0.363, 0.381, 0.157, and 0.028 μM, respectively.

**Figure 2:**
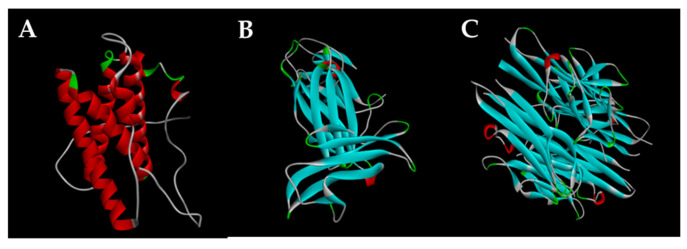
IL-6, IL-12 p40, and TNF-α proteins. (**A**) 2L3Y, (**B**) 1F42 and (**C**) 2AZ5.

**Figure 3:**
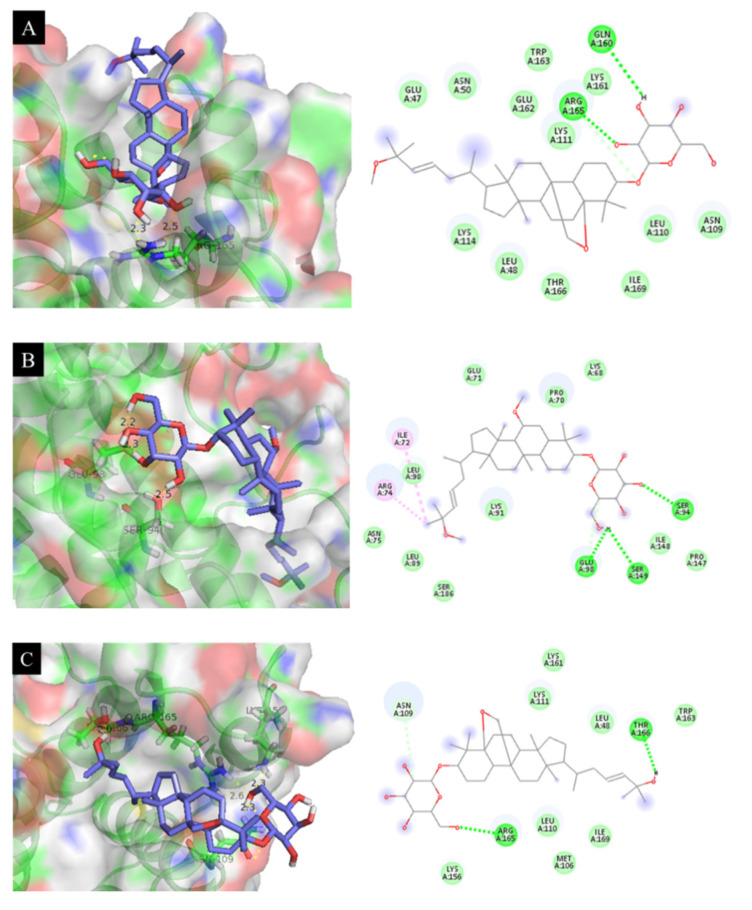

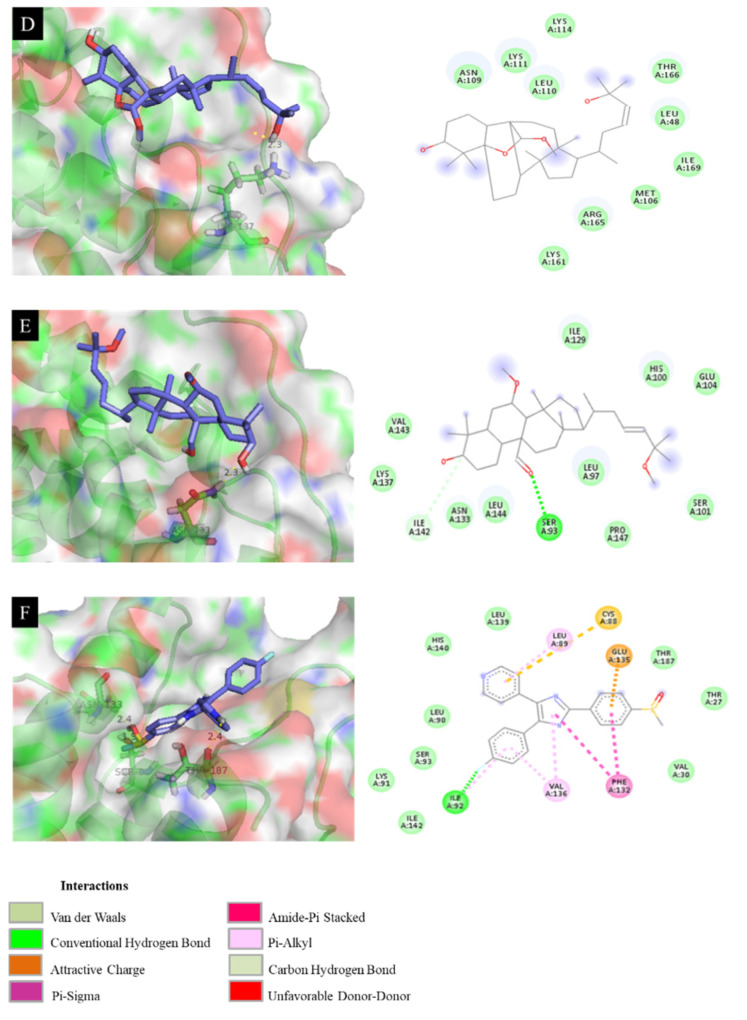
Docking simulation of the interactions between compounds **3** (**A**), **4** (**B**), **6** (**C**), **11 (D**), **12** (**E**), and **SB203580** (**F**), respectively, and the 2L3Y protein of IL-6 expression.

We demonstrated the docking of the active metabolites and **SB203580** into 2L3Y protein (Figure 2) using the PyRx 0.9.4 virtual screening software. Table 2 shows the docking score energy and root-mean-square deviation (RMSD) between the active compounds and protein with various interactions, including hydrogen bond interactions and the interaction distances between amino acids and the active sites. From the docking results, compounds **3**, **4**, **6**, **11**, and **12** had binding energies of −7.18, −7.48, −7.26, −6.99, and −6.41 kcal·mol^−1^, respectively. The corresponding ligand interactions of **3** and **4** with 2L3Y protein were hydrogen bond interactions between the enzyme residues and the hydroxyl groups in the sugar rings of the two compounds (Figure 3). As illustrated in Figure 3C, the ligand interactions of **6** with the IL-6 protein involved four hydrogen bonds with Lys156, Thr166, Arg165, and Asn109 with bond distances of 2.3, 2.0, 2.6, and 2.3 Å, respectively. These interactions corresponded to the hydrogen-bonding interactions between the enzyme residues and hydroxyl groups in both sugar rings and position C-25. Interestingly, the activities of compounds **11** and **12** were interpreted as hydrogen bonds with residues Lys137 and Asn133, respectively, with bond distances of 2.3 Å (Table 2). The corresponding ligand interactions of **11** and **12** with the protein involved hydrogen bonds between the enzyme residues and hydroxyl groups at C-25 and C-3, respectively (Figure 3). These findings suggest the important roles of the hydroxyl groups at positions C-3 and C-25 as well as the sugar rings in inhibition of IL-6 expression.

### 2.2. Inhibition of IL-12 Expression

IL-12 is a heterodimeric pro-inflammatory cytokine that was first recovered from EBV-transformed B cell lines [26]. It comprises a 35-kDa light chain (p35) and a 40-kDa heavy chain (p40). p35 is homologous with other single-chain cytokines, whereas p40 is homologous with the extracellular domain of members of the hematopoietic cytokine receptor family. The unusual structure of IL-12 might have evolved from a primordial cytokine in the IL-6 family and one of its receptors [27]. Recent evidence points to a critical role for IL-12 in the pathogenesis of rodent models of Th1-mediated autoimmune diseases, such as type-1 diabetes, rheumatoid arthritis, multiple sclerosis, and inflammatory bowel disease [28].

This study evaluated the inhibitory effects 15 isolated cucurbitane-type triterpenoids (**1**–**15**) on IL-12 p40 expression. Surprisingly, all 15 isolated compounds markedly inhibited IL-12 production with IC_50_ values of 0.012–1.360 μM, whereas the positive control **SB203580** had an IC_50_ of 3.500 μM (Table 1). Compounds **4**–**6**, **8**, **9**, **11**, and **12** had the greatest activity with IC_50_ values of 0.031, 0.063, 0.012, 0.085, 0.052, 0.073, and 0.045 μM, respectively. Subsequently, the most active compounds and positive control were docked with the 1F42 protein (Figure 2) of IL-12 p40 in the same grid. The docking results support the inhibition of IL-12 p40 expression by the most active compounds (**4**–**6**, **8**, **9**, **11**, and **12**), with binding energies of −6.93, −7.28, −7.34, −6.98, −7.02, −6.66, and −6.32 kcal·mol^−1^, respectively (Table 3).

**Figure 4:**
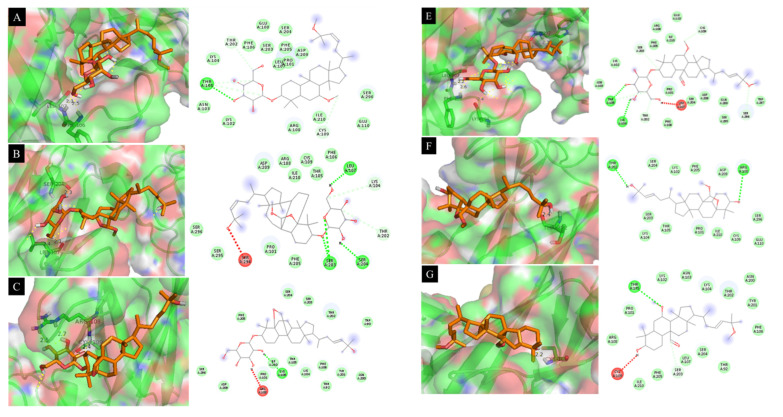

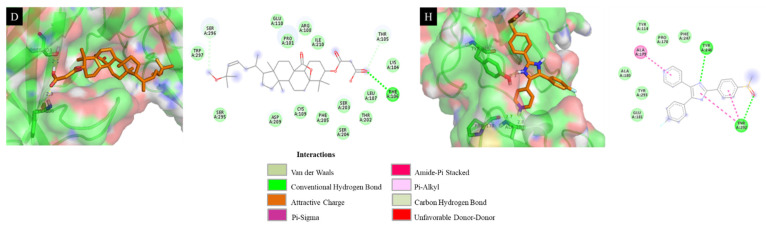
Docking simulation of the interactions between compounds **4**–**6** (**A**–**C**), **8** (**D**), **9** (**E**), **11** (**F**), **12** (**G**), and **SB203580** (**H**), respectively, and the 1F42 protein of IL-12 p40 expression.

All of the corresponding ligand interactions of terpenoids **4**–**6** and **9** with the 1F42 protein involved hydrogen bonds between the enzyme residues and hydroxyl groups in the sugar rings of these compounds (Figure 4). The great effect of compound **4** was due to two hydrogen bonds with residues Leu107 and Phe106 with bonding distances of 2.1 and 2.5 Å, respectively. Compound **5** formed two hydrogen bonds with residues Ser204 and Leu107 with respective bonding distances of 2.3 and 2.1 Å. The activity of compound **6** was interpreted as due to two hydrogen bonds with residues Arg108 and Cys109 with bond distances of 2.5 and 2.4 Å, respectively (Table 3). The activity of compound **9** resulted from the hydrogen bonds between enzyme residues Leu107, Phe106, and Lys104 and hydroxyl groups in the sugar rings, and between Trp297 and oxygen in the methoxy groups at C-25 (Figure 4E). Compound **8** formed two hydrogen bonds with residues Ser203 and Phe106 with two oxygen atoms of carboxylic acid in malonyl groups (Figure 4D). The activity of compound **11** resulted from hydrogen bonding between the hydroxyl group at C-25 and residue Thr202 with a small bond distance of 2.4 Å (Figure 4F). Compound **12** had a single hydrogen bond between residue Cys109 and the hydroxyl group at C-3 (Figure 4G). These data indicate the vital role of cucurbitane-type triterpenoids in the inhibition of IL-12 p40 production.

### 2.3. Inhibition of TNF-α Production

TNF-α is produced by macrophages during inflammation and can be activated by the endotoxin LPS. It is a multifunctional Th1 cytokine and one of the most important inflammatory cytokines. TNF-α plays a role in the pathogenesis of inflammatory diseases such as Crohn’s disease, rheumatoid arthritis, diabetes mellitus, and systemic lupus erythematosus [29,30].

As with their effects on IL-6 and IL-12 p40 expression, all of the isolated cucurbitane-type triterpenoids (**1**–**15**) inhibited TNF-α production with IC_50_ values of 0.033–4.357 μM, which are lower than the IC_50_ of 7.200 μM of the positive control **SB203580** (Table 1). Compounds **4**, **6**, **9**, and **11**–**14** had the greatest effect, with IC_50_ values of 0.810, 0.043, 0.087, 0.033, 0.142, 0.388, and 0.811 μM, respectively. These compounds were docked with 2AZ5 protein in the same grid (Figure 2). The docking results showed the remarkable effects of the most active compounds (**4**, **6**, **9**, and **11**–**14**) with binding energies of −8.23, −8.40, −8.22, −8.72, −7.83, −8.39, and −8.10 kcal·mol^−1^, respectively (Table 4).

**Figure 5:**
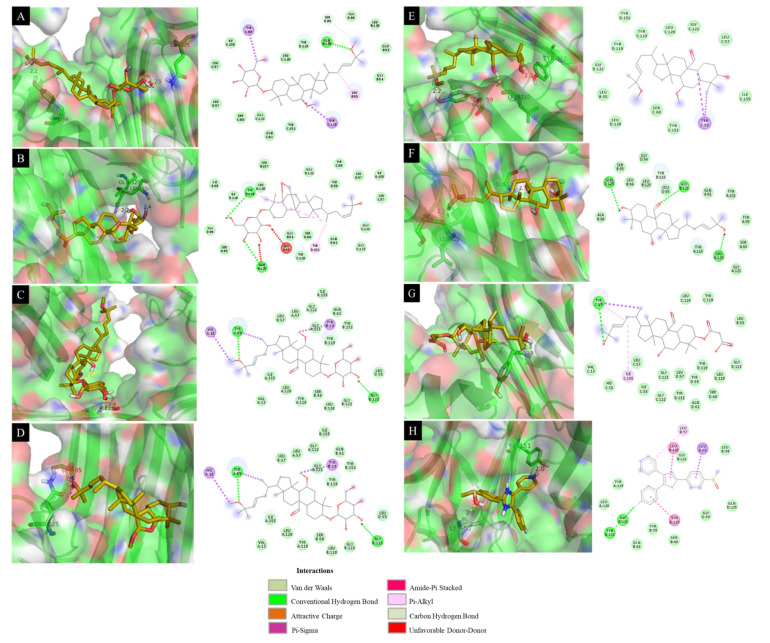
Docking simulation of the interactions between compounds **4** (**A**), **6** (**B**), **9** (**C**), **11**–**14** (**D**–**G**), and **SB203580** (**H**), respectively, and the 2AZ5 protein of TNF-α expression (**A**–**H**).

The interactions of **4**, **6**, and **9** with the TNF-α protein involved hydrogen bonds between enzyme residues and the hydroxyl groups in both sugar rings (Figure 5). As illustrated in Figure 5A, compound **4** formed three hydrogen bonds with the residues Tyr59, Gln125, and Gly121 with bond distances of 2.1, 2.5, and 2.2 Å, respectively. Compound **6** formed two hydrogen bonds with Leu55 and Gln125 of the 2AZ5 protein with bond distances of 2.4 and 2.7 Å, respectively (Figure 5B). Compound **9** formed a hydrogen bond with Gly121 with a bonding distance of 2.4 Å (Figure 5C). Compound **11** formed two hydrogen bonds between the hydroxyl group at C-25 and residues Ser95 and Gln125 with bond distances of 2.6 and 2.1 Å, respectively (Figure 5D). Compound **12** formed three hydrogen bonds between a hydroxyl group at C-25, oxygen in the methoxy group at C-25, and oxygen in the aldehyde group at C-19 and the residues Tyr151, Tyr59, and Leu120 with bond distances of 2.3, 2.2, and 2.6 Å, respectively (Figure 5E). The activity of compound **13** was based on bonding between a hydroxyl group at C-3 and oxygen in the methoxy group at C-25 and residues Gly121, Leu120, and Ser60 with bonding distances of 2.3, 1.9, and 2.5 Å, respectively (Figure 5F). Compound **14** formed a single bond between the hydroxyl group at C-25 and residue Tyr119 with a bond distance of 2.3 Å (Figure 5G). This demonstrates the roles of the hydroxyl groups at both C-3 and C-25, and the sugar rings, methoxy group at C-25, and the aldehyde group at C-19 in the inhibition of TNF-α expression.

In conclusion, 15 cucurbitane-type triterpenoids (**1**–**15**) were isolated from *M*. *charantia* fruit. Their structures were unambiguously established and their inhibitory effects on pro-inflammatory cytokine (IL-6, IL-12 p40, and TNF-α) production were characterized. The potential anti-inflammatory effects of the isolated triterpenoids (**1**–**15**) increase our understanding of the chemotaxonomic properties of the Cucurbitaceae, and the mechanisms underlying the anti-inflammatory properties of *M*. *charantia*. This is the first report of the inhibitory effects of isolated constituents of *M*. *charantia* fruit on the pro-inflammatory cytokines IL-6, IL-12 p40, and TNF-α. Based on this study and in silico simulations, we determined that the ligand likely docked in the receptor. Thus, compounds isolated from *M*. *charantia* fruit are potential candidates for treating inflammation and related diseases.

## 3. Materials and Methods

### 3.1. General Experimental Procedures

The optical rotation values were confirmed using a JASCO DIP-370 digital polarimeter (Hachioji, Tokyo, Japan). ESI mass spectra were obtained using an Agilent 1200 LC-MSD Trap spectrometer (Kyoto, Japan). LC-MS/MS analysis was performed by using a Shimadzu LCMS-8040 system (Kyoto, Japan) in positive and negative mode. NMR spectra were carried out on a JEOL ECA 400 and 600 spectrometer (JEOL Ltd., Tokyo, Japan) with TMS used as an internal standard. NMR data processing was recorded with the MestReNova 14.0 program. Sephadex LH-20 (Sigma-Aldrich, St. Louis, MO, USA), and Diaion HP-20 (Supelco™, Bellefonte, PA, USA) resins. Thin-layer chromatography (TLC) and YMC RP-18 resins were performed using pre-coated silica gel 60 F_254_ and RP-18 F_254S_ plates (both 0.25 mm, Merck, Darmstadt, Germany), and the spots were detected under UV light at 254, and 365 nm wavelengths and using 10% H_2_SO_4_, followed by heating for 3–5 min. The chemicals used were purchased from commercial suppliers and used as received. All chemical reagents were purchased from Sigma-Aldrich (St. Louis, MO, USA).

### 3.2. Plant Material

The fruits of *Momordica charantia* were obtained from an herbal company. Plant identification was verified by an expert botanist (Prof. Young Ho Kim). A representative specimen of the *M. charantia* (CNU MC1917) was conserved in the Herbarium of the Natural Product Laboratory, Chungnam National University, Daejeon, Korea.

### 3.3. Extraction and Isolation

The dried fruits of *M. charantia* (5.0 kg) were cut into pieces and extracted with MeOH (10 L × 3 times) at room temperature. The methanol solution was concentrated using a rotatory evaporator to give a residue of the MeOH extract (360 g), which was further suspended in H_2_O and successively partitioned with *n*-hexane (H), dichloromethane (CH_2_Cl_2_), and ethyl acetate (EtOAc) to afford H (300 g), D (35 g) EtOAc (E, 40 g), and aqueous extracts (W), respectively. Extract D was separated on silica gel using a mobile phase of CH_2_Cl_2_-MeOH (100:1–1:1, *v*/*v*) to afford eight fractions D1–D8, respectively. Fraction D3 was further separated into nine subfractions by YMC CC, eluted with a gradient of MeOH-H_2_O (1:3 to 1:0, *v*/*v*) to give four subfractions (D3A–D3D), respectively. Fraction D3C was further purified by YMC CC with MeOH-H_2_O (2:1–4:1, *v*/*v*) to obtain compounds **1** (6.6 mg), **2** (5.8 mg), **3** (9.5 mg), **4** (15.6 mg), and **5** (16.8 mg). Purification of the subfraction D3D via silica gel column eluted with EtOAc-MeOH-H_2_O (7:1:0.1, *v*/*v*/*v*) was further isolated by RP-C_18_ silica gel (MeOH-H_2_O, 3:1, 6:1, *v*/*v*) to yield compounds **7** (9.0 mg), **8** (4.9 mg), **11** (26.5 mg), and **15** (6.6 mg). Fraction D6 was isolated by RP-C_18_ silica gel eluted with (MeOH-H_2_O, 3:1, 6:1, *v*/*v*), and Sephadex™ LH-20 column using MeOH-H_2_O (5:1. *v*/*v*) to afford compounds **9** (7.2 mg), **10** (15.3 mg), **12** (27.8 mg), **13** (315.9 mg), **14** (3.0 mg), and **6** (24.0 mg).

#### Physical and the Key of Spectroscopic Data of New Natural Compound

Compound **1**. White amorphous powder, [α]_D_^20^ -77.8 (*c* = 0.2, MeOH); IR: ^1^H-NMR (400 MHz, CDCl_3_) *δ*_H_ 1.07 (s, 3H, H-26), 1.15 (s, 3H, H-26), 1.25 (s, 3H, H-26), 1.43 (s, 3H, H-26), 1.07 (s, 3H, H-26), 1.07 (s, 3H, H-26), 1.07 (s, 3H, H-26), 1.07 (s, 3H, H-26), 1.50 (s, 6H, H-26, and H-28), 5.71, 5.68 (OMe-3) and ^13^C-NMR (100 MHz, CDCl_3_): *δ*_C_ 21.7 (C-1), 29.9 (C-2), 80.0 (C-3), 41.7 (C-4), 146.1 (C-5), 121.5 (C-6), 75.1 (C-7), 50.6 (C-8), 36.9 (C-9), 22.7 (C-10), 29.4 (C-11), 29.4 (C-12), 45.7 (C-13), 48.2 (C-14), 34.9 (C-15), 27.7 (C-16), 50.1 (C-17), 15.0 (C-18), 208.0 (C-19), 36.5 (C-20), 18.9 (C-21), 39.5 (C-22), 128.5 (C-23), 137.1 (C-24), 75.6 (C-25), 30.8 (C-26), 30.8 (C-27), 26.2 (C-28), 27.3 (C-29), 18.2 (C-30), 56.1 (3-OMe), 51.2 (7-OMe), 50.3 (25-OMe). HR-ESI-MS: *m*/*z*: [M + Na]^+^ *m*/*z* 537.3918.

### 3.4. Cell Culture and Reagents

BMDCs were grown from wild-type C57BL/6 mice (Orient Bio Inc., Seoul, Korea) [31,32]. All animal procedures were approved by and performed according to the guidelines of the Institutional Animal Care and Use Committee of Jeju National University (#2016-0059). Briefly, bone marrow from the tibia and femur was obtained by flushing with Dulbecco’s Modified Eagle Medium (DMEM; Welgene, Gyeongsan, Korea) and bone marrow cells were cultured in RPMI-1640 medium containing 10% heat-inactivated fetal bovine serum (FBS; Gibco, New York, USA), 50 μM of 2-ME, and 2 mM of glutamine, supplemented with 3% J558L hybridoma cell culture supernatant containing the granulocyte-macrophage colony-stimulating factor (GM-CSF). The culture medium containing GM-CSF was replaced every other day. On day 6 of culturing, non-adherent cells and loosely adherent DC aggregates were harvested, washed, and resuspended in RPMI-1640, supplemented with 5% FBS. DCs were incubated in 48-well plates at a density of 1 × 10^5^ cells/0.5 mL and then treated with the isolated compounds at the indicated concentration for 1 h before stimulation with 10 ng/mL of LPS from *Salmonella minnesota* (Alexis, New York, NY, USA). Supernatants were harvested 18 h after stimulation. Concentrations of murine IL-12 p40, IL-6, and TNF-α in the culture supernatants were determined by ELISA (BD PharMingen, San Diego, CA, USA) following the manufacturer’s protocol [31,32]. All experiments were carried out at least three times. Data are presented as the mean and the standard deviation (SD) of three independent experiments.

### 3.5. Cytokine Production Measurements

The BMDCs were incubated in 48-well plates in 0.5 mL containing 1 × 10^5^ cells per well, and then treated with isolated compounds **1**−**15** at the indicated concentration for 1 h before stimulation with 10 ng/mL LPS from Salmonella Minnesota (Alexis, New York, NY, USA). Supernatants were collected 18 h after stimulation. Concentrations of murine IL-12 p40, IL-6, and TNF-α in the culture supernatants were identified by ELISA (BD PharMingen, San Diego, CA, USA) according to the manufacturer’s instructions.

The inhibitory activity (I) was expressed as the inhibition rate (%), which was calculated from the following formula:

(1) $$I=\frac{\mathrm{Cdvc}-\mathrm{Cdcc}}{\mathrm{Cdvc}}\times 100$$

Cdvc: Cytokine level (ng/mL) in vehicle-treated DCs; Cdcc: Cytokine level (ng/mL) in compound treated DC. The data was obtained by at least three independent experiments performed in triplicate.

### 3.6. Cell Viability Assay

To identify the effects of isolated compounds on cell viability, the MTT assay was carried out [33]. BMDCs were incubated with 1 to 50 µM of isolated compounds for 18 h. The results demonstrate that tested compounds (**1**−**15**) displayed no notable cytotoxicity against BMDCs.

### 3.7. Statistical Analysis

All results are presented as the means ± SD. Data were analyzed by one-factor analysis of variance (ANOVA). * *p*-value < 0.05, and ** *p*-value < 0.01 were considered statistically significant. All experiments were repeated at least three times independently.

### 3.8. Preparation of Structures of Proteins Molecular Docking

The structures of IL-6, IL-12 p40, and TNF-α proteins resolved using X-ray diffraction (resolution of 2.16 Å) were downloaded from the Worldwide Protein Data Bank (PDB: 2L3Y, 1F42, and 2AZ5). The crystal structure was prepared for docking using Discovery Studio Client (2020). This step mainly involved the addition of missing disulfide bonds, removal of water molecules, the addition of missing hydrogen atoms, filling of missing amino acids side chains, and optimization of hydrogen bonds. The PyRx 0.9.4 virtual screening software was then used for restrained minimization until the average RMSD of the non-hydrogen atoms converged to 0.30 Å. Thus, this step allowed sufficient movement of heavy atoms to relax strained bonds, angles, and clashes.

The 2D chemical structure (flat structure) of the isolated compounds and positive control were automatically converted to the 3D chemical structure (three-dimensional structure) by the ChemBioOffice 12.0 software. The energy of the generated compounds was then minimized using the PyRx 0.9.4 software.

### 3.9. Molecular Docking of Isolated Compounds with IL-6, IL-12 p40, and TNF-α Proteins

Docking of compounds into prepared protein via conducting the docking process with the PyRx 0.9.4 software was carried out by following the method of placing compound fragments into the triangle matching and retaining the eight best configurations of each compound in the bonding complex for further analysis. The grid for docking studies was generated to enclose all the residues of IL-6, IL-12 p40, and TNF-α proteins making polar interactions with the atoms of the positive control. The best configuration is the one with the lowest docking score (DS) energy (kcal.mol^–1^). This score is the total energy consumed for the formation of bonding interactions between the compounds and the protein selected.

### 3.10. Docking Results Analysis

Discovery Studio Client was used for the analysis of the interactions between the compounds and target protein, and the performance of interaction on 2D and 3D planes. Various interactions, such as hydrogen bonds, van der Waals interactions, π–anion bonds, π–π bonds, and the interaction distance between amino acids and the active sites of compounds were plotted. Van der Waals interactions are detected by contact with hydrophilic and hydrophobic surfaces between the compounds and their bonding point.

## Figures and Tables

**Table 1:** Inhibition of isolated cucurbitane-type triterpenoids (**1**–**15**) on IL-6, IL-12 p40, and TNF-α production in LPS-stimulated bone marrow-derived dendritic cells.

Compounds	IC_50_ (µM)		
	IL-6	IL-12 p40	TNF-α
**1**	0.886 ± 0.118	1.192 ± 0.104	2.809 ± 0.264
**2**	1.616 ± 0.211	0.733 ± 0.041	2.616 ± 0.181
**3**	0.245 ± 0.008	0.495 ± 0.003	4.357 ± 0.037
**4**	0.363 ± 0.015	0.031 ± 0.001	0.810 ± 0.068
**5**	0.582 ± 0.024	0.063 ± 0.017	2.144 ± 0.151
**6**	0.381 ± 0.039	0.012 ± 0.000	0.043 ± 0.006
**7**	1.299 ± 0.222	0.551 ± 0.048	3.888 ± 0.149
**8**	1.348 ± 0.052	0.085 ± 0.005	1.568 ± 0.209
**9**	0.568 ± 0.004	0.052 ± 0.005	0.087 ± 0.033
**10**	0.858 ± 0.002	0.553 ± 0.010	2.143 ± 0.003
**11**	0.157 ± 0.011	0.073 ± 0.086	0.033 ± 0.002
**12**	0.028 ± 0.005	0.045 ± 0.026	0.142 ± 0.009
**13**	1.962 ± 0.096	0.795 ± 0.086	0.388 ± 0.015
**14**	0.751 ± 0.038	0.299 ± 0.002	0.811 ± 0.005
**15**	1.553 ± 0.245	1.360 ± 0.101	3.716 ± 0.011
**SB203580** **^a^**	5.000 ± 0.080	3.500 ± 0.080	7.200 ± 0.060

^a^: Positive control.

**Table 2:** Docking simulation results with docking score energy (DS) and root-mean-square deviation (RMSD) between isolated compounds (**3**, **4**, **6**, **11**, and **12**) and the 2L3Y protein.

Compounds	DS (kcal·mol−1)	RMSD (Å)	Interaction with Amino Acid
**3**	−7.18	18.64	Arg 165 (2.3 Å)
**4**	−7.48	17.07	Ser 94 (2.5 Å), Glu 98 (2.2 Å)
**6**	−7.26	21.27	Lys 156 (2.3 Å), Thr 166 (2.0 Å), Arg 165 (2.6 Å), Asn 109 (2.3 Å)
**11**	−6.99	18.85	Lys 137 (2.3 Å)
**12**	−6.41	11.79	Asn 133 (2.3 Å)
**SB203580 ^a^**	−7.96	7.02	Asn 133 (2.4 Å), Ser 93 (1.9 Å), Thr 187 (2.4 Å)

^a^: Positive control.

**Table 3:** Docking simulation results with docking score energy (DS) and root-mean-square deviation (RMSD) between isolated compounds (**4**–**6**, **8**, **9**, **11**, and **12**) and the 1F42 protein.

Compounds	DS (kcal·mol^−1^)	RMSD (Å)	Interaction with Amino Acid
**4**	−6.93	12.15	Leu 107 (2.1 Å), Phe 106 (2.5 Å)
**5**	−7.28	5.67	Ser 204 (2.3 Å), Leu 107 (2.1 Å)
**6**	−7.34	13.21	Arg 108 (2.5 Å), Cys 109 (2.4 Å)
**8**	−6.98	11.93	Ser 203 (2.1 Å), Phe 106 (2.3 Å)
**9**	−7.02	13.86	Trp 297 (2.3 Å), Leu 107 (2.2 Å), Phe 106 (2.6 Å), Lys 104 (2.4 Å)
**11**	−6.66	16.38	Thr 202 (2.4 Å)
**12**	−6.32	10.39	Cys 109 (2.2 Å)
**SB203580 ^a^**	−7.41	11.14	Tyr 246 (2.3 Å), Pro 178 (2.7 Å), Glu 181 (2.8 Å)

^a^: Positive control.

**Table 4:** Docking simulation results with docking score energy (DS) and root-mean-square deviation (RMSD) between isolated compounds (**4**, **6**, **9**, and **11**–**14**) and the 2AZ5 protein.

Compounds	DS (kcal·mol^−1^)	RMSD (Å)	Interaction with Amino Acid
**4**	−8.23	10.86	Tyr 59 (2.1 Å), Gln 125 (2.5 Å), Gly 121 (2.2 Å)
**6**	−8.40	12.39	Leu 55 (2.4 Å), Gln 125 (2.7 Å)
**9**	−8.22	9.15	Gly 121 (2.4 Å)
**11**	−8.72	15.28	Ser 95 (2.6 Å), Gln 125 (2.1 Å)
**12**	−7.83	14.34	Tyr 151 (2.3 Å), Leu 120 (2.6 Å), Tyr 59 (2.2 Å)
**13**	−8.39	9.48	Gly 121 (2.3 Å), Leu 120 (1.9 Å), Ser 60 (2.5 Å)
**14**	−8.10	13.16	Tyr 119 (2.3 Å)
**SB203580 ^a^**	−8.22	14.53	Leu 55 (2.8 Å), Tyr 151 (2.0 Å)

^a^: Positive control.

## Data Availability

Not applicable.

## Sample Availability

Sample of the isolated compounds (**1**−**15**) available from the authors.
